# Precision diagnosis of *GABRA1*-associated encephalopathies and epilepsy: optimizing variants classification and molecular subregional effects

**DOI:** 10.3389/fgene.2026.1818471

**Published:** 2026-05-19

**Authors:** Wen-Hui Liu, Qiu-Li Li, Hai-Peng Li, Qian-Ru Wen, Si-Qi Zhang, Yan Ding, Heng Meng

**Affiliations:** 1 Department of Neurology, The First Affiliated Hospital and Clinical Neuroscience Institute of Jinan University, Guangzhou, China; 2 Department of Neurology, The First People’s Hospital of Chenzhou Affiliated to the University of South China, The First Affiliated Hospital of Xiangnan University, Chenzhou, China; 3 The Sixth Affiliated Hospital of Jinan University, Guangzhou, China

**Keywords:** epilepsy, GABRA1, missense variants, molecular subregions, pathogenicity assessment

## Abstract

**Background:**

*GABRA1* variants are associated with a broad spectrum of epileptic phenotypes ranging from mild idiopathic generalized epilepsy to severe developmental and epileptic encephalopathy (DEE). To date, the majority of the identified *GABRA1* variants are missense. Evaluating the pathogenicity of missense variants is a great challenge in genetics. This study aimed to explore reliable biological tools to optimize pathogenic classification of variants, thereby improving precision diagnosis of *GABRA1*-associated encephalopathies and epilepsy.

**Methods:**

The dataset of disease-associated and control *GABRA1* missense variants was curated. The location of these variants was visualized, to analyze the molecular subregional effects. The performance of 34 algorithms in evaluating the pathogenicity of *GABRA1* variants was systematically analyzed, including accuracy, sensitivity, specificity, positive predictive value (PPV), negative predictive value (NPV), Matthews correlation coefficient (MCC), F-score, and the area under the receiver operating characteristic curve (AUC).

**Result:**

A total of 61 GABRA1 missense variants were analyzed, including 30 pathogenic/likely pathogenic variants from patients with *GABRA1*-associated epilepsies and 31 benign/likely benign controls from the gnomAD database. The pathogenicity and phenotypes of these variants showed significant domain dependence: all transmembrane variants caused severe developmental and epileptic encephalopathy (DEE), the extracellular domain had the highest phenotypic heterogeneity, and the phenotype distribution differed significantly between functionally critical regions and other regions (P = 0.01), indicating a molecular subregional effect. We evaluated 34 commonly used algorithms, which varied considerably in performance. Ensemble and deep learning algorithms showed superior overall performance, with MetaLR and PrimateAI achieving the highest accuracy (0.9167) and AlphaMissense yielding the best AUC (0.9644). Tools like M-CAP and CADD_phred had low specificity. All tools except fathmm-XF showed highly significant score differences between groups (P < 0.0001), and high-performance tools presented a clear bimodal distribution with minimal overlap.

**Conclusion:**

Ensemble learning and deep learning algorithms are highly effective for predicting the pathogenicity of *GABRA1* missense variants. These computational tools provide reliable support for the pathogenicity assessment of *GABRA1* variants in clinical genetic diagnosis.

## 1 Introduction

The γ-aminobutyric acid type A (GABA_a_) receptor is the major inhibitory ionotropic receptor in the central nervous system, composed of multiple subunits including α, β, γ, and δ, among which the α1 subunit is encoded by the *GABRA1* gene (Johannesen et al., 2016). GABA_a_ receptors inhibit neuronal excitability and maintain neural network homeostasis by mediating Cl^−^ influx, thereby playing a pivotal role in regulating sleep, anxiety, and epileptic seizures (Carvill et al., 2014; Winsky-Sommerer, 2009).

Mutations in *GABRA1* have been extensively reported to be associated with a spectrum of epilepsy disorders, including developmental and epileptic encephalopathy 19 (DEE-19, OMIM∗ 615744) and the susceptibility genes of childhood absence epilepsy-4 (ECA-4, OMIM∗ 611136) and juvenile myoclonic epilepsy-5 (JME-5, OMIM∗ 611136); these phenotypes are often accompanied by intellectual disability, motor dysfunction, and behavioral abnormalities (Reinthaler et al., 2015) (Liu et al., 2023). Patients with *GABRA1* exhibited strong clinical heterogeneity. Some patients respond poorly to conventional antiepileptic drugs such as phenytoin or carbamazepine, leading to unfavorable prognosis (Ma et al., 2006). Among *GABRA1* gene variants, missense variants are the most common pathogenic type, accounting for more than 90% of reported mutations. These variants alter the secondary or tertiary structure, stability, or interaction with other subunits of the protein by substituting a single amino acid (Vanoye et al., 2014). For instance, missense variants located in transmembrane domains may affect ion channel function, whereas variants in extracellular loops may interfere with ligand-binding sites, resulting in loss-of-function or gain-of-function effects (Carvill et al., 2014; Fedele et al., 2018).

However, multiple challenges appeared in the clinical assessment of the variants pathogenicity. First, many variants are rare or occur *de novo*, lacking sufficient evidence of familial segregation. Second, the continuous spectrum of epileptic phenotypes from mild epilepsies to lethal encephalopathy makes quantification of functional effects difficult. In addition, population databases such as gnomAD show a large number of low-frequency variants annotated as variants of uncertain significance (VUS), which require multi-source evidence for reclassification according to the guidelines of the American College of Medical Genetics and Genomics (ACMG) (Richards et al., 2015). Traditional functional validation methods, such as electrophysiological recordings, can provide direct evidence but are limited by long experimental cycles, high costs, and inapplicability to large-scale screening. Therefore, reliable bioinformatics algorithms/indicators are urgently needed to bridge this gap (Hernandez and Macdonald, 2019).

Existing *in silico* prediction algorithms (e.g., SIFT, PolyPhen-2, MetaSVM, etc.) classify the pathogenicity of missense variants based on sequence conservation, protein structure, and evolutionary information, and have been widely used to optimize the diagnosis of hereditary diseases (Montanucci et al., 2024). These algorithms provide quantitative scores by calculating the potential impact of variants on protein stability and function, helping to distinguish benign from pathogenic variants. Nevertheless, their performance varies considerably across different genes and disease contexts, necessitating gene-specific evaluation and optimization.

Similarly, in the precision medicine framework for encephalopathies-associated variants in *CHD2* and *SCN3A*—common pathogenic genes for developmental and epileptic encephalopathies—diagnostic efficiency can be improved through algorithm optimization and/or analysis of molecular subregional effects, enabling effective assessment of the pathogenicity of missense variants (Tang et al., 2020; Gu et al., 2025; Wang et al., 2026). These findings provide possible methodological insights for the genetic diagnosis of epilepsy-related genes. Gene-specific evaluation for *GABRA1* may reveal gene-specific patterns and assist in guiding clinical decision-making.

In this study, we systematically collected a dataset of disease-associated missense variants and control variants of the *GABRA1* gene, evaluated the performance indicators of 34 commonly used algorithms, including accuracy, sensitivity, specificity, Matthews correlation coefficient (MCC), and F1-score, and compared their discriminative power via ROC curve analysis. Meanwhile, we explored the distribution of variants in protein subregions and their impact on algorithm analysis. We sought to identify reliable bioinformatics algorithms and indicators for assessing the pathogenicity of *GABRA1* variants, and to provide a reference for optimizing genetic diagnostic strategies. This may contribute to the development of precision medicine for epilepsy and support individualized patient management (Montanucci et al., 2024).

## 2 Materials and methods

### 2.1 Variants collection

To assess the ability of *in silico* tools to predict the pathogenicity of missense variants in *GABRA1*, variants were split into two groups: disease-associated variants and controls. Disease-associated variants were retrieved from the Human Gene Mutation Database (HGMD) and PubMed using the query: *GABRA1* AND (variant OR mutation). These variants were subsequently subjected to a stringent selection process, requiring them to meet the following criteria: (1) the disease diagnosis is supported by sufficient clinical information; (2) the involvement of other known disease-causing genes is largely excluded; (3) the origin of the variant can be explicitly determined; and (4) the pathogenicity of the variant has been evaluated by experts and met the ACMG criteria. Control variants were selected based on the following conditions: (1) the genotype is absent in the non-neurological control populations of gnomAD database; (2) the variants are non-neuro in gnomad; and (3) the minor allele frequency is less than 0.01 in both gnomAD v2 and v4 datasets, in order to prevent bias in the evaluation of algorithms that rely on population frequencies. The data review cutoff was November 1, 2025. To assess the ability of *in silico* tools to predict pathogenicity of missense variants in *GABRA1*, variants were initially split into two groups: disease-associated variants and controls. Disease-associated variants were retrieved from the Human Gene Mutation Database (HGMD) and PubMed using the query: *GABRA1* AND (variant OR mutation). They were filtered again with a stringent selection step, bringing in only those variants that had clinical information and explainable origins in genetic disease in the family. The control variants were retrieved from ClinVar and classified as “benign” or “likely benign”. Data review cutoff was November 1, 2025.

Molecular subregional effect analysis: A stratified approach was used to group variants by subregion (extracellular, transmembrane, cytoplasmic) (Hernandez and Macdonald, 2019; Macdo et al., 2010).

### 2.2 In silico prediction

A panel of 34 bioinformatics tools was selected for this study, chosen based on their widespread use and established effectiveness in predicting the functional impact of missense variants. The selection of these tools was based on primary criteria: (1) inclusion in the ACMG variant interpretation guidelines or recommendation by clinical genetics consortia, or availability in major variant annotation databases such as Franklin (franklin.genoox.com), or VarSome (varsome.com); (2) the availability of well-defined and recommended score thresholds for pathogenicity classification; (3) the ability to generate a valid score for at least 80% of the variants included in this study. All prediction scores were obtained from the dbNSFP databases (dbnsfp.org) to ensure reproducibility. These tools are summarized in Supplementary Table S1 (Adzhubei et al., 2010; Alirezaie et al., 2018; Brandes et al., 2023; González-Pérez and López-Bigas, 2011; Cheng et al., 2023; Choi et al., 2012; Dereli et al., 2024; Dong et al., 2015; Ioannidis et al., 2016; Jagadeesh et al., 2016; Kircher et al., 2014; Kumar et al., 2009; Li et al., 2022; Liu et al., 2020; Malhi et al., 2020; Pejaver et al., 2020; Quang et al., 2015; Raimondi et al., 2017; Reva et al., 2011; Rogers et al., 2018; Schwarz et al., 2010; Vaser et al., 2016).

This study adopted a two-step threshold strategy to serve two distinct, complementary research purposes, which eliminates any apparent inconsistency. First, literature-recommended default thresholds were used exclusively for the head-to-head benchmark comparison of all 34 algorithms. This unified general standard ensures the fairness, consistency and reproducibility of inter-algorithm performance evaluation, as different tools have inherently different scoring scales and cannot be directly compared using their respective gene-specific thresholds. Second, gene-specific optimal thresholds were derived subsequently for clinical application purposes. Generic default thresholds are designed for pan-gene use and often perform poorly in rare disease genes like *GABRA1*. For each top-performing algorithm identified via benchmarking, the optimal threshold was calculated using Youden’s Index (J = Sensitivity + Specificity - 1), which balances sensitivity and specificity specifically for GABRA1 variants. These thresholds can be used to more accurately calibrate ACMG PP3/BP4 evidence levels in clinical genetic diagnosis.

### 2.3 Evaluation of predictive performance

To evaluate predictive performance of these *in silico* tools, we obtained true positives (TP), true negatives (TN), false positives (FP), and false negatives (FN), and calculated accuracy, sensitivity, specificity, positive predictive value (PPV), negative predictive value (NPV), F-score, and the Matthews correlation coefficient (MCC). The F score was defined as $\frac{2\mathrm{PR}}{P+R}$ , where precision (P) = $\frac{TP}{TP+FP}$ and recall (R) = $\frac{TP}{TP+FN}$ . The Matthews correlation coefficient (MCC) score was calculated using the following equation $\frac{TP\times TN-FP\times FN}{\sqrt{\left(TP+FP\right)\left(TP+FN\right)\left(TN+FP\right)\left(TN+FN\right)}}$ , with the score ranging from −1 to 1; −1 indicates a completely wrong binary classifier, while one indicates a completely correct binary classifier.

ROC curves were then plotted for each tool as follows: disease-associated variants were used as the gold-standard positive samples and control missense variants with no relevant phenotypic as the gold-standard negative samples. Area under the curve (AUC) is reported.

### 2.4 Statistical analysis

All statistical analyses were done in R (4.5.1). For comparisons between two independent samples, the choice of statistical test was contingent upon the normality of the data. Normality was assessed using the Shapiro-Wilk test. If the data followed a normal distribution, Student’s t-test was employed. Conversely, for non-normally distributed data, the Mann-Whitney U test (or Wilcoxon rank-sum test) was utilized. A p-value <0.05 was considered statistically significant for all comparisons.

## 3 Results

### 3.1 Distribution and features of *GABRA1* missense variants

**FIGURE 1:**
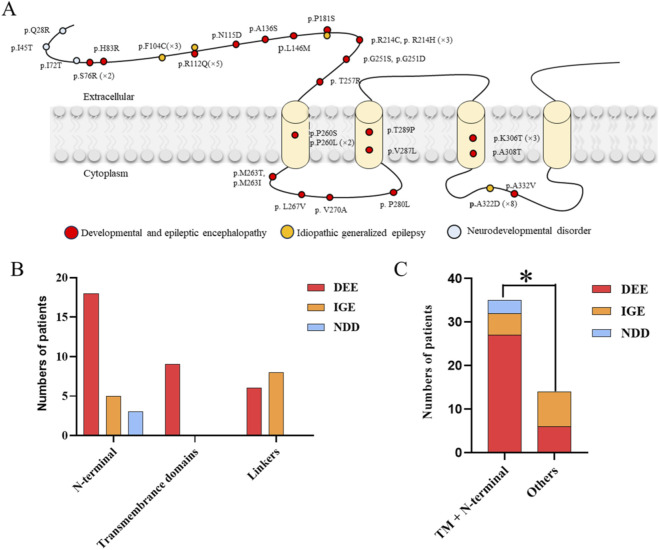
Distribution of *GABRA1* missense variants and correlation with clinical phenotypes. **(A)** Schematic of *GABRA1* missense variant loci across protein functional domains. **(B)** Bar plot showing the number of cases with different clinical phenotypes in each domain. **(C)** Comparison of phenotype distribution between functionally critical regions (N-terminal extracellular + transmembrane domains) and other regions (cytoplasmic/linker regions). The difference between groups was statistically significant (*P* = 0.01). Red: DEE; Yellow: IGE; Blue: NDD.

In this study, the dataset of missense variants in the *GABRA1* gene included 30 disease-associated variants (N = 30) and 31 control variants (N = 31). These variants were distributed across different functional regions of the protein, including the N-terminal extracellular domain and the four transmembrane domains. Variants in *GABRA1* are mainly associated with developmental and epileptic encephalopathy (DEE), idiopathic generalized epilepsy (IGE), and neurodevelopmental disorders (NDDs). Specifically, variants located in the extracellular domain included p.Q28R, p.I45T, p.I72T, p.H83R, p.S76R, p.F104C, p.R112Q, p.N115D, p.A136S, p.L146M, p.P181S, p.R214C, p.R214H, p.G251S, p.G251D, and p.T257R. Variants in the transmembrane domains included p.P260S, p.P260L, p.V287L, p.T289P, p.A308T, and p.K308T. Variants in the cytoplasmic domain included p.M263T, p.M263I, p.L267V, p.V270A, p.P280L, p.A322D, and p.A332V (Figure 1A; Supplementary Table S1).

Exploratory analysis of clinical phenotypes revealed that missense variants in *GABRA1* most frequently cause DEE, followed by IGE and NDD with comorbidity. Stratification by protein structural domain (Figure 1B) showed 18 cases of DEE, 5 cases of IGE and 3 cases of NDD in the N-terminal extracellular domain, all 9 cases in the transmembrane domain presented with the DEE phenotype, and the linker/cytoplasmic domains were dominated by 16 cases of DEE and 8 cases of IGE. When the variant location were divided into the “functionally critical regions” (encompassing the N-terminal extracellular domain and transmembrane domain) and “other regions” for comparative analysis, the results demonstrated that the clinical phenotypes corresponding to the functionally critical regions were significantly biased toward severe forms, mainly DEE with a small number of IGE and NDD cases, whereas the other regions were predominated by the milder IGE phenotype with almost no NDD cases, and the difference between the two groups was statistically significant (*P* = 0.01, Figure 1C). Notably, due to the limited number of variants per domain, these subregional associations are preliminary and require validation in larger independent cohorts.

### 3.2 Performance metrics of prediction algorithms

**FIGURE 2:**
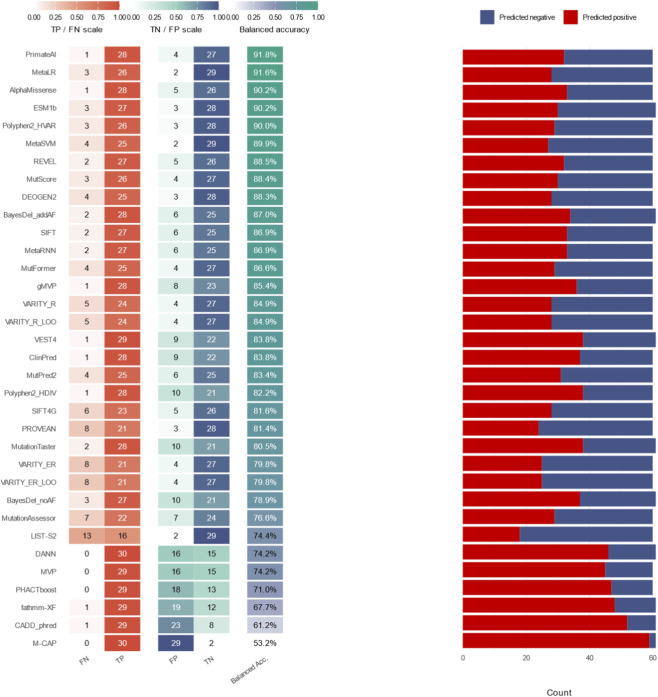
Performance evaluation of algorithms. For each tool, the left heat-map panel reports the rates of true positives (TP) and false negatives (FN) for pathogenic variants and true negatives (TN) and false positives (FP) for control variants. The middle column gives the resulting balanced accuracy (balanced acc.), which was used to rank the tools from top to bottom. The right stacked bar shows the proportion of variants each algorithm using recommended thresholds.

This study systematically evaluated the pathogenicity prediction performance of 34 bioinformatics tools for *GABRA1* missense variants using a final dataset of 61 variants consisting of 30 pathogenic variants from ClinVar, HGMD, and published epilepsy-related genetic studies as well as 31 benign control variants from the gnomAD database following rigorous curation; the analysis revealed significant differences in performance among the tools across various metrics (Figure 2; Supplementary Table S2), with accuracy ranging from 0.52 (M-CAP) to 0.9167 (MetaLR, PrimateAI), sensitivity from 0.55 (LIST-S2) to 1.0000 (MVP, PHACTboost, DANN, M-CAP), specificity from 0.06 (M-CAP) to 0.94 (MetaSVM, MetaLR, LIST-S2), positive predictive value (PPV) from 0.38 (M-CAP) to 0.9286 (MetaLR), negative predictive value (NPV) from 0.69 (LIST-S2) to 1.00 (MVP, PHACTboost, DANN, M-CAP), Matthews correlation coefficient (MCC) and F1-score ranking consistently with MetaLR and PrimateAI performing the best at 0.83 and 0.84 for MCC and 0.91 and 0.92 for F1-score respectively, and overall ensemble predictors such as MetaLR, MetaSVM, REVEL, and BayesDel_addAF together with deep-learning tools including AlphaMissense and ESM1b showing superior comprehensive performance while M-CAP, CADD_phred, DANN, and fathmm-XF exhibited obvious limitations in discriminating benign variants.

### 3.3 ROC curve analysis and AUC values

**FIGURE 3:**
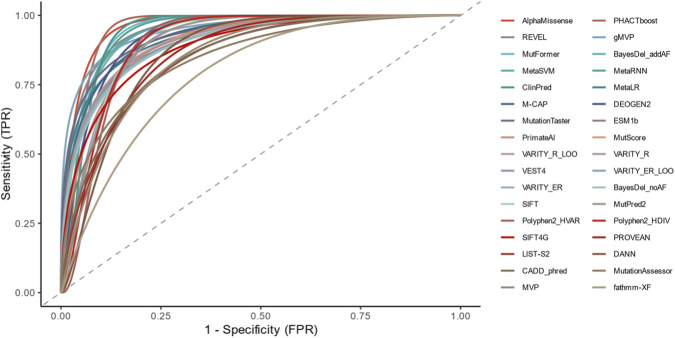
Receiver operating characteristic curve (ROC) performance and optimal thresholds of *in silico* tools for *GABRA1* variants. Combined ROC curves showed each color is one tool, and the dashed line meant no discrimination. All curves are provided in Supplementary Table S3.

**FIGURE 4:**
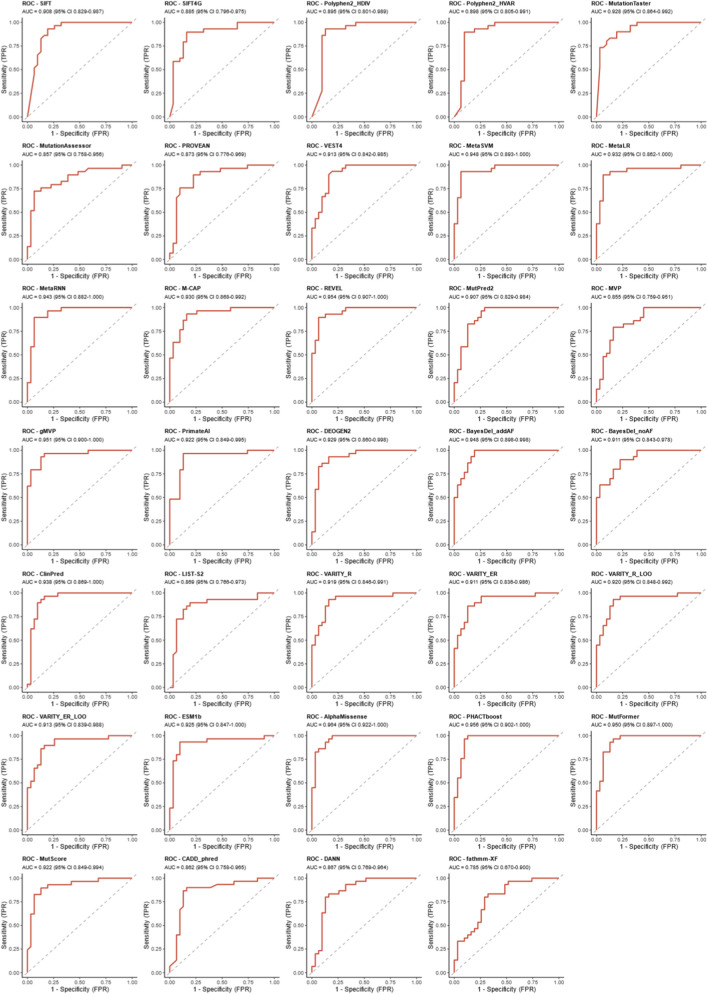
All ROC curves across 34 algorithms. ROC curves were presented with AUC and 90%CI, including SIFT, SIFT4G, Polyphen2_HDIV, Polyphen2_HVAR, MutationTaster, MutationAssessor, PROVEAN, VEST4, MetaSVM, MetaLR, MetaRNN, M-CAP, REVEL, MutPred2, MVP, gMVP, PrimateAI, DEOGEN2, BayesDel_addAF, BayesDel_noAF, ClinPred, LIST-S2, VARITY_R, VARITY_ER, VARITY_R_LOO, VARITY_ER_LOO, ESM1b, AlphaMissense, PHACTboost, MutFormer, MutScore, CADD_phred, DANN, and fathmm-XF.

To visually present and compare the predictive performance of each tool, receiver operating characteristic (ROC) curves were plotted in this study. The area under the curve (AUC), as a quantitative index of the ROC curve, can comprehensively reflect the diagnostic accuracy of a tool (Figures 3, 4; Supplementary Table S3). Consistent with the above results, ROC curve analysis showed that AlphaMissense (AUC = 0.9644) performed the best, followed by PHACTboost (0.9555), REVEL (0.9544), gMVP (0.9511), MutFormer (0.9499), BayesDel_addAF (0.9484), MetaSVM (0.9477), MetaRNN (0.9433), and ClinPred (0.9377), while fathmm-XF (AUC = 0.7849) had the lowest AUC value (Figure 2). These AUC values indicate that ensemble and deep-learning tools such as AlphaMissense, PHACTboost, REVEL, gMVP, BayesDel_addAF, and MetaSVM exhibit excellent discriminative ability for pathogenic variants and control variants.

### 3.4 Between-group comparison of score distributions

**FIGURE 5:**
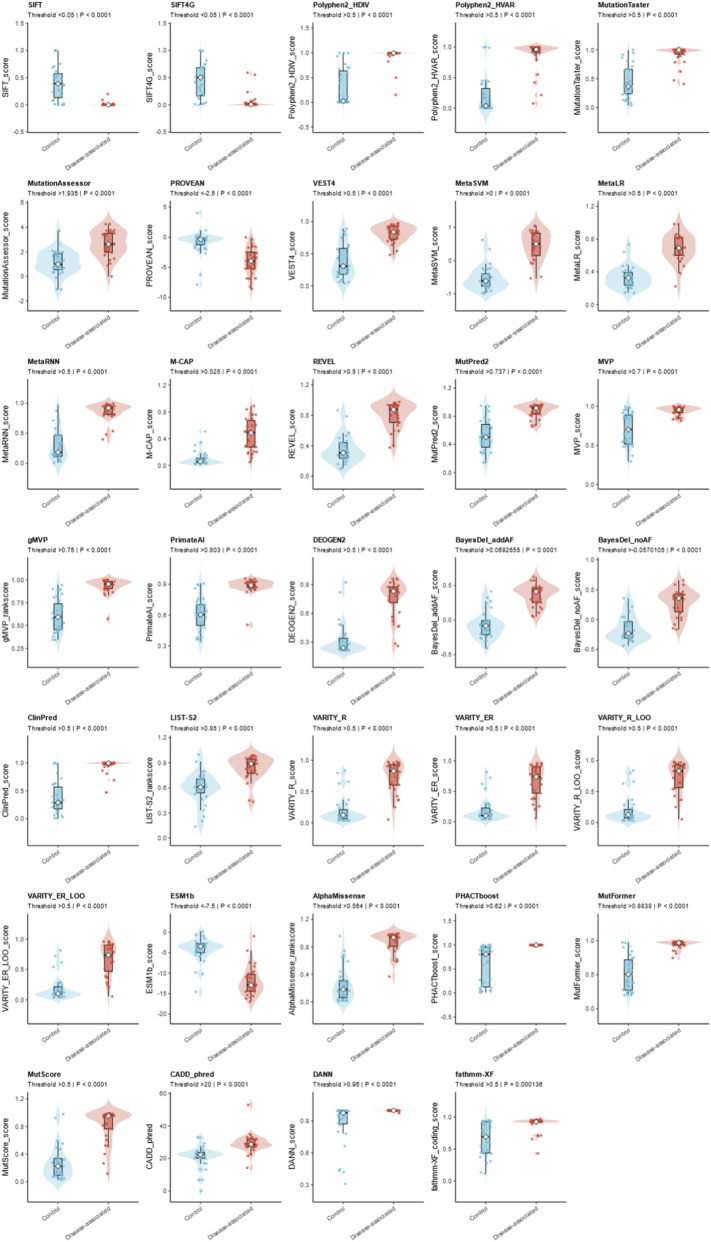
Grid of thirty-six violin plots compares bioinformatic prediction scores between two groups labeled controls and disease-associated, across various metrics such as SIFT, Polyphen, Mutation Taster, and CADD. In each plot, disease-associated variants (red) consistently show higher prediction scores than controls (blue), with significant P values indicated for each metric. Boxplots are overlaid within each violin plot, and threshold values are stated in plot subtitles.

In this study, we predicted continuous scores from 34 *in silico* prediction tools, classified variants into “pathogenic” and “control” groups using predefined binary cutoff thresholds, and visualized the score distribution of each tool using combined violin and scatter plots (Figure 5). The results demonstrated that all tools except fathmm-XF showed highly significant between-group differences in prediction scores between pathogenic and control variants (P < 0.0001), with fathmm-XF alone yielding a between-group difference p-value of 0.000136. Among these tools, the scores of SIFT, SIFT4G, PolyPhen2_HDIV, PolyPhen2_HVAR, MetaLR, MetaSVM, REVEL, ClinPred, AlphaMissense, and PHACTboost exhibited a clear bimodal distribution pattern with nearly no overlap between the pathogenic and control groups, highlighting their excellent discriminative ability between *GABRA1* pathogenic and benign control variants. In contrast, tools including M-CAP, CADD_phred, DANN, and fathmm-XF presented a markedly broader and continuous score distribution with substantial numerical overlap between the two groups, indicating the limited discriminative capacity of these tools and reflecting significant performance differences across distinct prediction algorithms for the pathogenicity classification of *GABRA1* missense variants.

## 4 Discussion

The *GABRA1* gene encodes the α1 subunit of the GABA_a_ receptor, a key molecule mediating inhibitory neurotransmission in the central nervous system. Missense variants in this gene are associated with a continuous phenotypic spectrum ranging from benign idiopathic generalized epilepsy (IGE) to severe developmental and epileptic encephalopathy (DEE). Constrained by the high proportion of rare *de novo* variants, insufficient familial segregation evidence, and the long cycle and low throughput of traditional functional experiments, a large number of variants of uncertain significance (VUS) persist in clinical practice, significantly hindering the precise diagnosis of *GABRA1*-associated encephalopathies and epilepsy. In this study, we systematically evaluated the performance of 34 commonly used bioinformatics algorithms and comprehensively analyzed the association between variant molecular subregions and clinical phenotypes. We aimed to establish a standardized variant interpretation framework tailored to the characteristics of the *GABRA1* gene and provide empirical evidence for improving the accuracy and clinical utility of genetic diagnosis.

In terms of pathogenicity prediction algorithms, our results demonstrated significant divergence in the discriminative power of different tools for GABRA1 missense variants, with ensemble learning algorithms and deep learning algorithms exhibiting the best overall performance. The area under the curve (AUC) values of AlphaMissense, PHACTboost, REVEL, BayesDel_addAF, and MetaSVM all exceeded 0.94, and the accuracy of MetaLR and PrimateAI reached 0.9167, showing stable and reliable ability to distinguish pathogenic from benign variants. This trend is consistent with previous studies on epilepsy-related pathogenic genes such as *CHD2* and *SCN3A*, suggesting that ensemble strategies have cross-gene general applicability in the interpretation of rare disease gene variants. The advantage of ensemble algorithms lies in their ability to integrate multidimensional information including sequence conservation, protein spatial structure, evolutionary constraint strength, and population allele frequency, effectively compensating for the information bias of single algorithms. For example, BayesDel_addAF, which incorporates allele frequency annotations, had significantly fewer false positives than BayesDel_noAF without frequency information, making it particularly valuable for *GABRA1*, where rare mutations are the predominant type. In contrast, tools such as M-CAP, fathmm-XF_coding, and CADD_phred had low specificity and were prone to misclassifying benign variants as pathogenic. Their limitations may stem from over-reliance on sequence conservation features and failure to fully reflect the structure-function constraints and gene-specific functional landscape of ion channel genes. The outstanding performance of deep learning algorithms such as AlphaMissense, ESM1b, and MutFormer in this study is closely related to their training on large-scale protein structure data and evolutionary information, indicating that deep learning models still have room for further optimization and clinical application in specific gene contexts such as *GABRA1*. Notably, as acknowledged in the Limitations section, potential circularity bias due to overlapping training and test datasets may slightly inflate the absolute performance values reported here, but the comparative ranking of tools and the identified optimal algorithm combination remain valid for *GABRA1* variant interpretation.

At the molecular subregional level, our exploratory analysis suggested that the pathogenicity and phenotypic severity of *GABRA1* missense variants may exhibit domain dependence. In our cohort, all variants in the transmembrane domain were associated with the severe DEE phenotype, which is consistent with the known role of this region as the core functional domain of the GABA_a_ receptor for ion channel gating. Larger cohorts are needed to confirm this association and quantify the exact risk of severe phenotypes associated with variants in different domains. The transmembrane domain directly forms the pore of the chloride channel and participates in gating regulation and ion selectivity maintenance. Amino acid substitutions in this region can significantly disrupt channel opening, closing, and ion permeability, leading to severe inhibitory signal deficits and severe clinical phenotypes. The N-terminal extracellular domain had the largest number of variants and the most prominent phenotypic heterogeneity, encompassing severe DEE, IGE, and neurodevelopmental disorder (NDD) phenotypes. This is related to the diverse functions of this domain in mediating ligand binding, receptor subunit assembly, and allosteric regulation: variants located in the ligand-binding pocket can significantly impair GABA binding capacity, while variants at non-critical sites have milder effects on receptor affinity, ultimately forming a phenotypic spectrum of varying severity. Variants in the cytoplasmic and linker regions were predominantly associated with moderate phenotypes, with a high proportion of IGE and very few cases of severe NDD, suggesting that these regions have relatively mild effects on the core function of the receptor. Statistical analysis after dividing the N-terminal extracellular domain and transmembrane domain into functionally critical regions showed a significant difference in phenotypic distribution between functionally critical and non-critical regions (*P* = 0.01). This domain-phenotype association pattern is consistent with the classic pathogenic mechanism of ion channelopathies and can provide an intuitive reference for clinicians to quickly judge pathogenicity tendency and prognostic risk.

Based on the results of algorithm evaluation and subregional effect analysis, this study proposes a hierarchical interpretation strategy integrating algorithm optimization, gene-specific thresholds, and molecular subregional information to improve diagnostic efficiency and reduce the proportion of VUS. First, a multi-algorithm consensus voting mechanism should be adopted, prioritizing the combination of high-performance tools such as MetaSVM, BayesDel_addAF, and PrimateAI. Consistent judgments by at least two tools can be used as supporting evidence for pathogenicity, reducing false positives and false negatives while maintaining high sensitivity. Second, the molecular subregional location should be incorporated into the interpretation process with differential weights: variants in the transmembrane domain have strong associations with pathogenicity and severe phenotypes, so the weight of algorithm predictions can be increased and functional validation should be prioritized; variants in the extracellular domain have high phenotypic heterogeneity, and deep learning tools are recommended to capture subtle pathogenic effects; variants in the cytoplasmic/linker regions require comprehensive judgment combined with strict clinical phenotypes and familial evidence. In addition, the multi-population allele frequency data integrated by BayesDel_addAF helps reduce European population bias, which may make it more suitable for genetic diagnosis in East Asian populations and improve the accuracy of local clinical interpretation after further validation.

## 5 Limitation

This study has several important limitations. First, the small sample size of 61 *GABRA1* missense variants included in this study may compromise the statistical robustness. Second, the small dataset limits the stability of the reported p-values and confidence intervals. Third, potential circularity bias exists as most algorithms were trained on ClinVar and HGMD, which overlap with our gold-standard variants and may overestimate performance. We used strict criteria to reduce this bias, retaining only variants with functional validation or clear segregation. While residual bias remains and absolute values need caution, the relative algorithm ranking is reliable for clinical use. Fourth, this study lacks experimental validation and relies only on *in silico* and retrospective data. Fifth, as a retrospective computational study, it has no prospective clinical verification. The proposed framework is not ready for routine clinical use and needs further validation in large clinical cohorts.

## 6 Conclusion

Through algorithm performance evaluation and molecular subregional effect analysis, this study demonstrates that ensemble learning and deep learning algorithms exhibit relatively superior performance in the determination of *GABRA1* missense variants, and the domain where the variant is located can serve as an important reference for phenotypic and pathogenicity judgment. The integrated application of optimized algorithms, gene-specific thresholds, and domain stratification information has the potential to improve the genetic diagnostic accuracy of *GABRA1-*associated encephalopathies and epilepsy, and may contribute to optimizing clinical interpretation processes, reducing the proportion of variants of uncertain significance (VUS), and supporting individualized patient management in the future. Notably, all findings are based on retrospective computational analysis, and their actual clinical effectiveness requires verification through prospective multi-center studies.

## Data Availability

The complete raw dataset generated and analyzed in this study, including detailed specifications of data format, variable definitions and table structure, is publicly available in the GitHub repository at https://github.com/wliu91868-hub/GABRA1-Data. All other relevant data supporting the findings of this study are available from the corresponding author upon reasonable request.

## References

[B1] Adzhubei I. A. Schmidt S. Peshkin L. Ramensky V. E. Gerasimova A. Bork P. (2010). A method and server for predicting damaging missense mutations. Nat. Methods 7 (4), 248–249. 10.1038/nmeth0410-248 20354512 PMC2855889

[B2] Alirezaie N. Kernohan K. D. Hartley T. Majewski J. Hocking T. D. (2018). ClinPred: prediction tool to identify disease-relevant nonsynonymous single-nucleotide variants. Am. J. Hum. Genet. 103 (4), 474–483. 10.1016/j.ajhg.2018.08.005 30220433 PMC6174354

[B3] Brandes N. Goldman G. Wang C. H. Ye C. J. Ntranos V. (2023). Genome-wide prediction of disease variant effects with a deep protein language model. Nat. Genet. 55 (9), 1512–1522. 10.1038/s41588-023-01465-0 37563329 PMC10484790

[B4] Carvill G. L. Weckhuysen S. McMahon J. M. Hartmann C. Møller R. S. Hjalgrim H. (2014). GABRA1 and STXBP1: novel genetic causes of Dravet syndrome. Neurology 82 (14), 1245–1253. 10.1212/WNL.0000000000000291 24623842 PMC4001207

[B5] Cheng J. Novati G. Pan J. Bycroft C. Žemgulytė A. Applebaum T. (2023). Accurate proteome-wide missense variant effect prediction with AlphaMissense. Science 381 (6664), eadg7492. 10.1126/science.adg7492 37733863

[B6] Choi Y. Sims G. E. Murphy S. Miller J. R. Chan A. P. (2012). Predicting the functional effect of amino acid substitutions and indels. PLoS One 7 (10), e46688. 10.1371/journal.pone.0046688 23056405 PMC3466303

[B7] Dereli O. Kuru N. Akkoyun E. Bircan A. Tastan O. Adebali O. (2024). PHACTboost: a phylogeny-aware pathogenicity predictor for missense mutations *via* boosting. Mol. Biol. Evol. 41 (7), msae136. 10.1093/molbev/msae136 38934805 PMC11251492

[B8] Dong C. Wei P. Jian X. Gibbs R. Boerwinkle E. Wang K. (2015). Comparison and integration of deleteriousness prediction methods for nonsynonymous SNVs in whole exome sequencing studies. Hum. Mol. Genet. 24 (8), 2125–2137. 10.1093/hmg/ddu733 25552646 PMC4375422

[B9] Fedele L. Newcombe J. Topf M. Gibb A. Harvey R. J. Smart T. G. (2018). Disease-associated missense mutations in GluN2B subunit alter NMDA receptor ligand binding and ion channel properties. Nat. Commun. 9 (1), 957. 10.1038/s41467-018-02927-4 29511171 PMC5840332

[B10] González-Pérez A. López-Bigas N. (2011). Improving the assessment of the outcome of nonsynonymous SNVs with a consensus deleteriousness score, condel. Am. J. Hum. Genet. 88 (4), 440–449. 10.1016/j.ajhg.2011.03.004 21457909 PMC3071923

[B11] Gu Y. J. Wang P. Y. Fu Q. Q. Lai J. H. Chen X. Liu X. H. (2025). Epilepsy-associated CHD2 missense variants and optimization strategies for genetic diagnosis: a comparative analysis of algorithms. Front. Neurol. 16, 1729387. 10.3389/fneur.2025.1729387 41383239 PMC12689356

[B12] Hernandez C. C. Macdonald R. L. (2019). A structural look at GABA(A) receptor mutations linked to epilepsy syndromes. Brain Res. 1714, 234–247. 10.1016/j.brainres.2019.03.004 30851244

[B13] Ioannidis N. M. Rothstein J. H. Pejaver V. Middha S. McDonnell S. K. Baheti S. (2016). REVEL: an ensemble method for predicting the pathogenicity of rare missense variants. Am. J. Hum. Genet. 99 (4), 877–885. 10.1016/j.ajhg.2016.08.016 27666373 PMC5065685

[B14] Jagadeesh K. A. Wenger A. M. Berger M. J. Guturu H. Stenson P. D. Cooper D. N. (2016). M-CAP eliminates a majority of variants of uncertain significance in clinical exomes at high sensitivity. Nat. Genet. 48 (12), 1581–1586. 10.1038/ng.3703 27776117

[B15] Johannesen K. Marini C. Pfeffer S. Møller R. S. Dorn T. Niturad C. E. (2016). Phenotypic spectrum of GABRA1: from generalized epilepsies to severe epileptic encephalopathies. Neurology 87 (11), 1140–1151. 10.1212/WNL.0000000000003087 27521439

[B16] Kircher M. Witten D. M. Jain P. O'Roak B. J. Cooper G. M. Shendure J. (2014). A general framework for estimating the relative pathogenicity of human genetic variants. Nat. Genet. 46 (3), 310–315. 10.1038/ng.2892 24487276 PMC3992975

[B17] Kumar P. Henikoff S. Ng P. C. (2009). Predicting the effects of coding non-synonymous variants on protein function using the SIFT algorithm. Nat. Protoc. 4 (7), 1073–1081. 10.1038/nprot.2009.86 19561590

[B18] Li C. Zhi D. Wang K. Liu X. (2022). MetaRNN: differentiating rare pathogenic and rare benign missense SNVs and InDels using deep learning. Genome Med. 14 (1), 115. 10.1186/s13073-022-01120-z 36209109 PMC9548151

[B19] Liu X. Li C. Mou C. Dong Y. Tu Y. (2020). dbNSFP v4: a comprehensive database of transcript-specific functional predictions and annotations for human nonsynonymous and splice-site SNVs. Genome Med. 12 (1), 103. 10.1186/s13073-020-00803-9 33261662 PMC7709417

[B20] Liu W. H. Luo S. Zhang D. M. Lin Z. S. Lan S. Li X. (2023). *De novo* GABRA1 variants in childhood epilepsies and the molecular subregional effects. Front. Mol. Neurosci. 16, 1321090. 10.3389/fnmol.2023.1321090 38269327 PMC10806124

[B21] Ma S. Blair M. A. Abou-Khalil B. Lagrange A. H. Gurnett C. A. Hedera P. (2006). Mutations in the GABRA1 and EFHC1 genes are rare in familial juvenile myoclonic epilepsy. Epilepsy Res. 71 (2-3), 129–134. 10.1016/j.eplepsyres.2006.06.001 16839746

[B22] Macdonald R. L. Kang J. Q. Gallagher M. J. (2010). Mutations in GABAA receptor subunits associated with genetic epilepsies. J. Physiol. 588 (11), 1861–1869. 10.1113/jphysiol.2010.186999 20308251 PMC2901974

[B23] Malhis N. Jacobson M. Jones S. J. M. Gsponer J. (2020). LIST-S2: taxonomy based sorting of deleterious missense mutations across species. Nucleic Acids Res. 48 (W1), W154–w161. 10.1093/nar/gkaa288 32352516 PMC7319545

[B24] Montanucci L. Brünger T. Boßelmann C. M. Ivaniuk A. Pérez-Palma E. Lhatoo S. (2024). Evaluating novel *in silico* tools for accurate pathogenicity classification in epilepsy-associated genetic missense variants. Epilepsia 65 (12), 3655–3663. 10.1111/epi.18155 39440667 PMC11647429

[B25] Pejaver V. Urresti J. Lugo-Martinez J. Pagel K. A. Lin G. N. Nam H. J. (2020). Inferring the molecular and phenotypic impact of amino acid variants with MutPred2. Nat. Commun. 11 (1), 5918. 10.1038/s41467-020-19669-x 33219223 PMC7680112

[B26] Quang D. Chen Y. Xie X. (2015). DANN: a deep learning approach for annotating the pathogenicity of genetic variants. Bioinformatics 31 (5), 761–763. 10.1093/bioinformatics/btu703 25338716 PMC4341060

[B27] Raimondi D. Tanyalcin I. Ferté J. Gazzo A. Orlando G. Lenaerts T. (2017). DEOGEN2: prediction and interactive visualization of single amino acid variant deleteriousness in human proteins. Nucleic Acids Res. 45 (W1), W201–w206. 10.1093/nar/gkx390 28498993 PMC5570203

[B28] Reinthaler E. M. Dejanovic B. Lal D. Semtner M. Merkler Y. Reinhold A. (2015). Rare variants in γ-aminobutyric acid type A receptor genes in rolandic epilepsy and related syndromes. Ann. Neurol. 77 (6), 972–986. 10.1002/ana.24395 25726841

[B29] Reva B. Antipin Y. Sander C. (2011). Predicting the functional impact of protein mutations: application to cancer genomics. Nucleic Acids Res. 39 (17), e118. 10.1093/nar/gkr407 21727090 PMC3177186

[B30] Richards S. Aziz N. Bale S. Bick D. Das S. Gastier-Foster J. (2015). Standards and guidelines for the interpretation of sequence variants: a joint consensus recommendation of the American college of medical genetics and genomics and the association for molecular pathology. Genet. Med. 17 (5), 405–424. 10.1038/gim.2015.30 25741868 PMC4544753

[B31] Rogers M. F. Shihab H. A. Mort M. Cooper D. N. Gaunt T. R. Campbell C. (2018). FATHMM-XF: accurate prediction of pathogenic point mutations *via* extended features. Bioinformatics 34 (3), 511–513. 10.1093/bioinformatics/btx536 28968714 PMC5860356

[B32] Schwarz J. M. Rödelsperger C. Schuelke M. Seelow D. (2010). MutationTaster evaluates disease-causing potential of sequence alterations. Nat. Methods 7 (8), 575–576. 10.1038/nmeth0810-575 20676075

[B33] Tang B. Li B. Gao L. D. He N. Liu X. R. Long Y. S. (2020). Optimization of *in silico* tools for predicting genetic variants: individualizing for genes with molecular sub-regional stratification. Brief. Bioinform 21 (5), 1776–1786. 10.1093/bib/bbz115 31686106

[B34] Vanoye C. G. Gurnett C. A. Holland K. D. George A. L. Kearney J. A. (2014). Novel SCN3A variants associated with focal epilepsy in children. Neurobiol. Dis. 62, 313–322. 10.1016/j.nbd.2013.10.015 24157691 PMC3877720

[B35] Vaser R. Adusumalli S. Leng S. N. Sikic M. Ng P. C. (2016). SIFT missense predictions for genomes. Nat. Protoc. 11 (1), 1–9. 10.1038/nprot.2015.123 26633127

[B36] Wang P. Y. Zhao J. X. Liu W. H. Chen Y. J. Wang H. W. (2026). Toward precision medicine in SCN3A variants-associated encephalopathies and epilepsy: optimizing genetic diagnosis and molecular subregional effects. Front. Neurol. 17, 1772239. 10.3389/fneur.2026.1772239 41725720 PMC12916406

[B37] Winsky-Sommerer R. (2009). Role of GABAA receptors in the physiology and pharmacology of sleep. Eur. J. Neurosci. 29 (9), 1779–1794. 10.1111/j.1460-9568.2009.06716.x 19473233

