# Supplementary material for: Precision diagnosis of GABRA1-associated encephalopathies and epilepsy: optimizing variants classification and molecular subregional effects
Source: Front Genet. 2026 May 19;17:1818471. doi: 10.3389/fgene.2026.1818471 (PMC13225776; doi:10.3389/fgene.2026.1818471)
Supplement: Supplementary file 3 [file Table1.docx]

**Table S1.** Characteristics of previously reported cases with *GABRA1* mutations.

| **Gender** | **Variants** | **MAF** | **Originated** | **Onset-age** | **Seizure type** | **Frequency of seizure** | **Therapy** | **Neurodevelopment** | **EEG** | **MRI** | **Diagnosis** | **Ref** |
| --- | --- | --- | --- | --- | --- | --- | --- | --- | --- | --- | --- | --- |
| NA | c.84G>T/p.Gln28His | *-/-* | *DN* | NA | NA | NA | NA | NA | NA | NA | NDD | PMID: 36619507 |
| NA | c.134T>C/p.Ile45Thr | *-/-* | *DN* | NA | NA | NA | NA | NA | NA | NA | NDD | PMID: 36619507 |
| NA | c.215T>C/p.Ile72Thr | *-/-* | *DN* | NA | NA | NA | NA | NA | NA | NA | JME | PMID: 37993639 |
| M | c.226A>C/p.Ser76Arg | *-/-* | *DN* | 5 mo | FoS, SE, fbTCS, GTCS | NA | Refractory | Severe GDD, speech delay, and behavioral problems | Blateral or multifocal paroxysmal activity | Normal | DEE  (DS-like) | PMID: 27521439 |
| F | c.226A>C/p.Ser76Arg | *-/-* | *DN* | 6 mo | GTCS, MS, AS | NA | Refractory | Severe GDD, speech delay, and autistic features | Generalized spike-waves, intermittent photic stimulation response | Normal | DEE  (DS) | PMID: 27521439 |
| NA | c.248A>G/p.His83Arg | *-/-* | *DN* | NA | NA | NA | NA | NA | NA | NA | DEE | PMID: 29186148 |
| M | c.311G>A/p.Phe104Cys | *-/-* | CS | 13 yr | MS | Rarely | VPA  (Sz - free) | Normal | Generalized spike-waves | Normal | IGE (JME) | PMID: 27521439 |
| M | c.311G>A/p.Phe104Cys | *-/-* | CS | 7. 5 yr | GTCS | Rarely | VPA, LTG  (Sz - free) | Normal | Generalized spike-waves generalized intermittent photic stimulation response | Normal | IGE  (GTCA) | PMID: 27521439 |
| F | c.311G>A/p.Phe104Cys | *-/-* | CS | 14 yr | GTCS | Rarely | NA (Sz - free) | Normal | Generalized spike-waves generalized intermittent photic stimulation response | Normal | IGE  (GTCA) | PMID: 27521439 |
| M | c.335G>A/p.Arg112Gln | *-/-* | *DN* | 11 mo | FS, GTCS, AS, fbTCS, MS | NA | NA | Moderate GDD | Generalized spike-wave | Normal | DEE  (DS) | PMID: 27521439 |
| F | c.335G>A/p.Arg112Gln | *-/-* | *DN* | 7 mo | GTCS, FS | Rarely | LEV (Sz - free) | GDD and Speech delay | Normal | Chiari I malfoemation | DEE  (DS-like) | PMID: 27521439 |
| M | c.335G>A/p.Arg112Gln | *-/-* | *DN* | 11 mo | FS, fbTCS, AS, SE, GTCS | NA | NA | Moderate GDD | Focal discharges | Calcified subependymal nodule in left lateral ventricle | DEE  (DS) | PMID: 27521439 |
| NA | c.335G>A/p.Arg112Gln | *-/-* | *DN* | 10 mo | FS, GTCS, atonic | NA | VPA, CLB  (Sz - free) | Mild GDD and Speech delay | Symmetrical θ wave | Normal | DEE  (DS) | PMID: 26918889 |
| F | c.335G>A/p.Arg112Gln | *-/-* | *DN* | 8 mo | Clonic | NA | LEV (Sz - free) | Normal | Normal | Normal | IGE  (GTCA) | PMID: 27521439 |
| F | c.343A>G/p.Asn115Asp | *-/-* | *DN* | 6 mo | fbTCS, FS, GTCS | 3-5 times / 1 wk | NA | Moderate GDD, behavioral problems | Multifocal and bilateral spikes | Normal | DEE  (DS) | PMID: 27521439 |
| M | c.406G>T/p.Ala136Ser | *-/-* | *DN* | NA | NA | NA | NA | NA | NA | NA | DEE (JME) | PMID: 34469436 |
| M | c.436C>A/p.Leu146Met | *-/-* | *DN* | 7 mo | FoS, fbTCS | every wks then mos | NA | Severe GDD | Multifocal paroxysmal activity | NA | DEE  (DS-like) | PMID: 27521439 |
| F | c.541C>T/p.Pro181Ser | *-/-* | *DN* | 12 yr | AS | NA | LTG, LEV (Sz - free for 13yr) | Mild-moderate GDD | NA | Normal | IGE  (JAE) | PMID: 31568673 |
| F | c.541C>T/p.Pro181Ser | *-/-* | *DN* | 8 yr | AS, atonic, GTCS | Daily | Refractory | Moderate GDD | Interictal:generalized polyspike waves and 2.5Hz spike waves  ictal: recorded absence seizures accompanied by generalized polyspikes and 2.5-3Hz spike wave activity. | Normal | DEE  (LGS) | PMID: 31568673 |
| F | c.640C>T/p.Arg214Cys | *-/-* | *DN* | 11 yr | FoS, GTCS, AS | 1 time / 1-2 mos | VPA, CZP (Remission) | GDD, ASD | Rhythmic generalized and bi-posterior quadrant spike and wave and polyspike and wave discharges which were time-locked with eyelid myoclonia | Periventricular leukomalacia and macrocephaly secondary to non-progressive ventriculomegaly | DEE | PMID: 31707987 |
| M | c.641G>A/p.Arg214His | *-/-* | *DN* | 8 mo | FS, GTCS | NA | Refractory | Severe GDD | Focal spike wave | NA | DEE  (DS) | PMID: 35937053 |
| M | c.641G>A/p.Arg214His | *-/-* | *DN* | 5 mo | FS, GTCS, FoS | NA | VPA, TPM (Remission) | Moderate GDD | Multifocal discharges, generalized spike-waves | NA | DEE  (DS) | PMID: 35937053 |
| F | c.641G>A/p.Arg214His | *-/-* | *DN* | 15 mo | FS, MS, GTCS, atonic, FoS | NA | Refractory | Severe GDD | Generalized spike-waves, bifrontal paroxysmal activity | Normal | DEE  (LGS) | PMID: 27521439 |
| F | c.751G>A/p.Gly251Ser | *-/-* | *DN* | 8 mo | FoS, GTCS, fbTCS, SE | NA | NA | Mild GDD | Normal | Normal | DEE  (DS) | PMID: 27521439 |
| M | c.752G>A/p.Gly251Asp | *-/-* | *DN* | 9 mo | FoS, | NA | VPA, LEV (Sz - free) | Speech delay | Slow background activity | NA | DEE | PMID: 27521439 |
| F | c.770C>G/p.Thr257Arg | *-/-* | *DN* | 4 mo | Spasms | NA | Refractory | Severe GDD and speech delay | Hypsarrhythmia, burst suppression, multifocal discharges | Normal | DEE  (WS) | PMID: 35937053 |
| F | c.778C> T/p.Pro260Ser | *-/-* | *DN* | 3 mo | Spasms, FoS, GTCS | NA | Refractory | Severe GDD | Hypsarrhythmia, burst suppression, multifocal discharges, generalized polyspike-wave | Normal | DEE  (WS) | PMID: 35937053 |
| F | c.779C>T/p.Pro260Leu | *-/-* | *DN* | 3.5 mo | Tonic, Spasms, MS | NA | VGB  (Remission) | Severe GDD, motor delay, speech delay and severe ID | Multifocal sharp-and-slow waves; hypsarrhythmia | Cerebral shrinkage due to ACTH therapy | DEE  (OS) | PMID: 26918889 |
| F | c.779C>T/p.Pro260Leu | *-/-* | *DN* | 1 mo | Spasms | NA | LTG  (Sz - free 3yr) | Severe GDD, motor delay, speech delay and severe ID | Diffuse spike-waves and polyspike-waves, hypsarrhythmia | Mild frontal lobe atrophy | DEE  (WS) | PMID: 26918889 |
| M | c.788T>C/p.Met263Thr | *-/-* | *DN* | 1 day | MS, Spasms | NA | Refractory | Severe GDD, motor delay, speech delay and severe ID | Multifocal sharp wave；hypsarrhythmia | Cerebral atrophy | DEE  (WS) | PMID: 26918889 |
| F | c.789G>C/p.Met263Ile | *-/-* | *DN* | 6 mo | Spasms | NA | ACTH  (Sz - free) | Severe GDD motor delay, speech delay and severe ID | Diffuse slow spike and waves, focal spikes at electrode on midline parietal region; hypsarrhythmia | Normal | DEE  (WS) | PMID: 26918889 |
| M | c.799C>G/p.Leu267Val | *-/-* | *DN* | NA | NA | NA | NA | NA | NA | NA | DEE | PMID: 39439447 |
| F | c.809T> C/p.Val270Ala | *-/-* | *DN* | 5 mo | FS, GTCS, FoS | NA | VPA, TPM, CZP (Remission) | Moderate GDD | Focal spike wave | Normal | DEE  (DS) | PMID: 35937053 |
| NA | c.839C>T/p.Pro280Leu | *-/-* | *DN* | NA | NA | NA | NA | NA | NA | NA | NDD | PMID: 36619507 |
| M | c.859G>T/p.Val287Leu | *-/-* | *DN* | 1 day | GTCS | NA | GBP  (Remission) | Severe GDD, motor delay, speech delay and severe ID | General slow background activity with multifocal spikes (7 mo); suppression burst (10 yr) | Cerebral, brain stem and cerebellar atrophy, thin corpus callosum | DEE | PMID: 26918889 |
| F | c.865A>C/p.Thr289Pro | *-/-* | *DN* | 1 day | Clonic, GTCS, FoS | Daily | Refractory | Severe GDD | Burst suppression, multifocal paroxysmal activity | Severe delayed myelination | DEE  (OS) | PMID: 27521439 |
| M | c.917A>C/p.Lys306Thr | *-/-* | *DN* | 8 mo | MS, GTCS | NA | Refractory | GDD, behavioral problems | Generalized spike-waves, bifrontal spike-wave, generalized intermittent photic stimulation response | Normal | DEE  (MAE) | PMID: 27521439 |
| M | c.917A>C/p.Lys306Thr | *-/-* | *DN* | 8 mo | AS, SE, FS, GTCS | NA | NA | Mild GDD | Generalized spike-wave, multifocal discharges, photoparoxysmal response | Normal | DEE  (DS) | PMID: 27521439 |
| NA | c.917A>C/p.Lys306Thr | *-/-* | *DN* | NA | NA | NA | NA | NA | NA | NA | DEE  (DS) | PMID: 24623842 |
| NA | c.922G > A/p.Ala308Thr | *-/-* | Maternal | NA | NA | NA | NA | NA | NA | NA | DEE | PMID: 40665309 |
| NA | c.965C>A/p.Ala322Asp | *-/-* | CS | 13 yr | MS, GTCS | NA | NA | NA | Generalized spike-wave, polyspike-and-wave discharges | NA | IGE  (JME) | PMID: 11992121 |
| NA | c.965C>A/p.Ala322Asp | *-/-* | CS | 14 yr | MS, GTCS | NA | NA | NA | Generalized spike-wave, polyspike-and-wave discharges | NA | IGE  (JME) | PMID: 11992121 |
| NA | c.965C>A/p.Ala322Asp | *-/-* | CS | 12 yr | MS, GTCS | NA | NA | NA | Generalized spike-wave, polyspike-and-wave discharges | NA | IGE  (JME) | PMID: 11992121 |
| NA | c.965C>A/p.Ala322Asp | *-/-* | CS | 8 yr | MS, GTCS, AS | NA | NA | NA | Generalized spike-wave, polyspike-and-wave discharges | NA | IGE  (JME) | PMID: 11992121 |
| NA | c.965C>A/p.Ala322Asp | *-/-* | CS | 13 yr | MS, GTCS | NA | NA | NA | Generalized spike-wave, polyspike-and-wave discharges | NA | IGE  (JME) | PMID: 11992121 |
| NA | c.965C>A/p.Ala322Asp | *-/-* | CS | 16 yr | MS, GTCS, AS | NA | NA | NA | Generalized spike-wave, polyspike-and-wave discharges | NA | IGE  (JME) | PMID: 11992121 |
| NA | c.965C>A/p.Ala322Asp | *-/-* | CS | 13 yr | MS, GTCS, AS | NA | NA | NA | Generalized spike-wave, polyspike-and-wave discharges | NA | IGE  (JME) | PMID: 11992121 |
| NA | c.965C>A/p.Ala322Asp | *-/-* | CS | 5 yr | MS, GTCS, AS | NA | NA | NA | Generalized spike-wave, polyspike-and-wave discharges | NA | IGE  (JME) | PMID: 11992121 |
| M | c.995C>T/p.Ala332Val | *-/-* | *DN* | 2 mo | FoS | NA | TPM, LTG, PGB (Sz - free) | Severe GDD and motor delay | NA | A similar picture of a cerebral brain atrophy, especially in the frontal and temporal regions. | DEE | PMID: 32047208 |
|  |  |  |  |  |  |  |  |  |  |  |  |  |

F, female; M, male; ASD, autism spectrum disorder; AS, absence seizures; ACTH, adrenocorticotropic hormone; BZDs, benzodiazepines; DEX, dexamethasone; CNZ, clonazepam; CLB. clobazam; CZP, clonazepam; d, day; CS, Co-segregated; DN, De novo; DS. Dravet syndrome; DEE, developmental and epileptic encephalopathy; EEG, electroencephalography; EMA, eyelid myoclonia with or without absences; FS, febrile seizures; FoS, focal seizures; fbTCS, focal to bilateral tonic clonic seizure; GBP, gabapentin; GDD, global development delay; GTCA, generalized tonic-clonic seizures alone; GTCS, generalized tonic-clonic seizures; IGE, idiopathic generalized epilepsy; JAE, juvenile absence epilepsy; JME, juvenile myoclonic epilepsy; LGS, [Lennox-Gastaut syndrome](https://pubmed.ncbi.nlm.nih.gov/29124439/); LTG: lamotrigine; LEV, levetiracetam; MRI, magnetic resonance imaging; MS, myoclonic seizure; mo, month; MAE, myoclonic absence epilepsy; MAF, minor allele frequency; NA, not available; PE, partial epilepsy; OS, Ohtahara syndrome; OXC, oxcarbazepine; POLE, photosensitive occipital lobe epilepsy; PGB, pregabalin; SE, status epilepticus; Sz - free，seizure - free; TPM. topiramate; VGB, vigabatrin; VPA, valproate; WS, West syndrome; wk: week; yr, year; Pz, electrode on midline parietal region.
